# Experimental dataset from a central composite design with two qualitative independent variables to develop high strength mortars with self-compacting properties

**DOI:** 10.1016/j.dib.2021.107738

**Published:** 2021-12-21

**Authors:** Lino Maia

**Affiliations:** a CONSTRUCT-LABEST, Faculty of Engineering (FEUP), University of Porto, Rua Dr. Roberto Frias, Porto 4200-465, Portugal; b Faculty of Exact Sciences and Engineering, University of Madeira, Campus da Penteada, Funchal 9020-105, Portugal

**Keywords:** Cement, Design of experiments, Mortar, Normensand, Response model, Superplasticiser

## Abstract

Fresh and hardening properties of cement-based materials are key factors for correctly choosing the constituent materials and their mix proportions. To optimize design-based mortar compositions for specific applications, response models are frequently applied to data collected from scientific approaches. Here, experimental dataset regarding to a design of experiments carried out in mortars through a central composite design with five independent variables is presented. Among the five independent variables, four were quantitative ones: Water_v_/Cement_v_, Superplasticyzer_m_/Powder_v_, Water_v_/Powder_v_, Sand_v_/Mortar_v_. The other independent variable was a qualitative one: Superplasticiser A or Superplasticiser B. In total 60 mortar compositions were done: for each qualitative variable a 2^4^ factorial design comprising of 16 treatment combinations enlarged by 8 axial runs plus 6 central runs, resulting in a central composite design with 30 mortar trial mix compositions. The following dependent variables were tested: the D-flow and the t-funnel to evaluate the fresh properties and the compressive at the age of 24 h and at the age of 28 days to evaluate the hardened properties. Based on this dataset, response models can be applied to find optimized mix compositions, with the effect of the two qualitative variables being determined.

**Specifications Table**

**Table:** 

Subject	Civil and Structural Engineering.
Specific subject area	Construction materials, concrete technology, high strength concrete, self-compacting concrete, mortars with self-compacting properties.
Type of data	Tables.
How the data were acquired	Data was acquired through readings in laboratory tests. The design of experiments (mortar mix compositions) was defined based on a central composite design. Then, after mixing the mortar workability was assessed by the flow and the V-funnel tests and the compressive strength was measured at the ages of 24 h and 28 days.
Data format	Raw.
Description of data collection	Just after mixing the time in the V-funnel was done and immediately after the slump test was done. Just after these tests, five prismatic specimens 4 × 4 × 16 [cm] were moulded and stored in a climatic chamber at 20 °C. At the age 23.5 h all the specimens were unmoulded. Three specimens were mechanically tested to achieve the compressive strength at the age of 24 h (with 15 min tolerance) and the remaining two specimens were stored in a climatic chamber at 20 °C and at least 98% of RH. These two specimens were mechanically tested to achieve the compressive strength at the age of 28 d Note that, the tensile strength test (without recording the value) was done previously to the compressive strength test in order to obtain two halves per specimen. Immediately after that, the compressive strength was done for each half of specimen.
Data source location	• Institution: Faculty of Civil Engineering of the University of Porto • City: Porto • Country: Portugal
Data accessibility	With the article.

**Value of the Data**

• Typical data published in papers based in a Central Composite Design applied to mortar mix compositions has no more than 20 experiments. The design of experiments is based in three independent variables. Here, the total volume of sand was an independent variable, too. That means an extra independent variable, therefore increasing the number of experiments for 30 experiments.
• Besides, two superplasticizers were used as qualitative independent variables. That leads to double the size of the experiments and allows analyses based on qualitative variables which are barely found in the literature.
• The mechanical properties are usually measured for one age. Here, the compressive strength was measured at the age of 24 h and at the age of 28 d. Thus, analyses both at early and later ages are possible.
• With a total of 60 mix composition testes (30 from each Central Composite Design regarding to a distinct superplasticiser), the outliers/errors analysis and statistical analysis are statistically better verified and surpassed by the large number of experiments. Then, the next coming response models are expected to be more precise.
• Researchers that are working/developing/applying/validating models related to mortars with self-compacting properties and/or with high strength may be interested in this data.
• This data may be applied to develop response models to study the effect of the constituent materials in the fresh and hardened properties. Researchers that are dealing with designing approaches of mix compositions with artificial intelligence may be interested in this data, too.

## 1 Data Description

Table 1 establishes the equivalence between the coded and the real values.

**Table 1:** Equivalence between the coded and real values.

Level	−2	−1	0	1	2
Water_v_/Cement_v_	0.78741	0.84110	0.89478	0.94847	1.00216
Water_v_/Powder_v_	0.02069	0.02210	0.02351	0.02492	0.02633
Water_v_/Powder_v_	0.46929	0.50129	0.53328	0.56528	0.59728
Sand_v_/Mortar_v_	0.42240	0.45120	0.48000	0.50880	0.53760

Table 2 displays the dataset for the mix proportions and the corresponding coded values of the mortars prepared for 1.61 L. The proportions regard to a design of experiments based on a central composite design for four independent variables. The dataset base includes 30 mixes. However, due to using qualitative variable – two superplasticizers – a second one central composite design of 30 mixes was added. Note: in Table 2, the total water added to the mixes was adjusted to the water effective plus the water to saturate the aggregates minus the water included in the superplasticiser.

**Table 2:** Proportions from the mortars produced.

Run Order	Water_v_/ Cement_v_	Water_v_/ Powder_v_	Water_v_/ Powder_v_	Sand_v_/ Mortar_v_	Cement [g]	Filler [g]	Superplasticiser A [g]	Superplasticiser B [g]	Sand [g]	Water Added [g]
1	0	0	0	0	1000.04	590.43	12.7237		2014.79	287.06
2	1	1	−1	1	855.56	664.74	13.0117		2135.68	260.37
3	1	−1	1	−1	1033.85	610.39	12.3647		1893.90	314.58
4	1	−1	−1	1	855.56	664.74	11.5387		2135.68	261.25
5	−1	−1	−1	1	964.78	569.61	11.5387		2135.68	261.25
6	1	−1	−1	−1	955.89	742.69	12.8917		1893.90	290.41
7	−1	−1	1	−1	1165.83	495.44	12.3647		1893.90	314.58
8	1	1	1	−1	1033.85	610.39	13.9432		1893.90	313.63
9	−1	1	−1	1	964.78	569.61	13.0117		2135.68	260.37
10	0	0	0	0	1000.04	590.43	12.7237		2014.79	287.06
11	1	−1	1	1	925.34	546.33	11.0669		2135.68	282.88
12	1	1	−1	−1	955.89	742.69	14.5375		1893.90	289.42
13	−1	1	1	1	1043.47	443.44	12.4797		2135.68	282.03
14	−1	−1	−1	−1	1077.92	636.41	12.8917		1893.90	290.41
15	0	0	0	0	1000.04	590.43	12.7237		2014.79	287.06
16	−1	1	−1	−1	1077.92	636.41	14.5375		1893.90	289.42
17	−1	−1	1	1	1043.47	443.44	11.0669		2135.68	282.88
18	1	1	1	1	925.34	546.33	12.4797		2135.68	282.03
19	−1	1	1	−1	1165.83	495.44	13.9432		1893.90	313.63
20	0	0	0	0	1000.04	590.43	12.7237		2014.79	287.06
21	2	0	0	0	892.89	683.75	12.7237		2014.79	287.06
22	−2	0	0	0	1136.40	471.66	12.7237		2014.79	287.06
23	0	2	0	0	1000.04	590.43	14.2506		2014.79	286.14
24	0	−2	0	0	1000.04	590.43	11.1969		2014.79	287.98
25	0	0	0	0	1000.04	590.43	12.7237		2014.79	287.06
26	0	0	2	0	1075.17	466.44	12.2139		2014.79	309.05
27	0	0	−2	0	918.36	725.22	13.2779		2014.79	263.15
28	0	0	0	2	889.26	525.03	11.3143		2256.57	256.66
29	0	0	0	−2	1110.81	655.83	14.1331		1773.02	317.46
30	0	0	0	0	1000.04	590.43	12.7237		2014.79	287.06
31	0	0	0	0	1000.04	590.43		16.9650	2014.79	282.82
32	1	1	−1	1	855.56	664.74		17.3489	2135.68	256.03
33	1	−1	1	−1	1033.85	610.39		16.4862	1893.90	310.46
34	1	−1	−1	1	855.56	664.74		15.3849	2135.68	257.40
35	−1	−1	−1	1	964.78	569.61		15.3849	2135.68	257.40
36	1	−1	−1	−1	955.89	742.69		17.1890	1893.90	286.11
37	−1	−1	1	−1	1000.04	590.43		16.9650	2014.79	282.82
38	1	1	1	−1	1165.83	495.44		16.4862	1893.90	310.46
39	−1	1	−1	1	1033.85	610.39		18.5909	1893.90	308.98
40	0	0	0	0	964.78	569.61		17.3489	2135.68	256.03
41	1	−1	1	1	925.34	546.33		14.7559	2135.68	279.19
42	1	1	−1	−1	955.89	742.69		19.3833	1893.90	284.58
43	−1	1	1	1	1000.04	590.43		16.9650	2014.79	282.82
44	−1	−1	−1	−1	1043.47	443.44		16.6396	2135.68	277.87
45	0	0	0	0	1077.92	636.41		17.1890	1893.90	286.11
46	−1	1	−1	−1	1077.92	636.41		19.3833	1893.90	284.58
47	−1	−1	1	1	1043.47	443.44		14.7559	2135.68	279.19
48	1	1	1	1	925.34	546.33		16.6396	2135.68	277.87
49	−1	1	1	−1	1000.04	590.43		16.9650	2014.79	282.82
50	0	0	0	0	1165.83	495.44		18.5909	1893.90	308.98
51	2	0	0	0	892.89	683.75		16.9650	2014.79	282.82
52	−2	0	0	0	1136.40	471.66		16.9650	2014.79	282.82
53	0	2	0	0	1000.04	590.43		19.0008	2014.79	281.39
54	0	−2	0	0	1000.04	590.43		14.9292	2014.79	284.24
55	0	0	0	0	1000.04	590.43		16.9650	2014.79	282.82
56	0	0	2	0	1075.17	466.44		16.2853	2014.79	304.98
57	0	0	−2	0	918.36	725.22		17.7039	2014.79	258.73
58	0	0	0	2	889.26	525.03		15.0858	2256.57	252.89
59	0	0	0	−2	1110.81	655.83		18.8442	1773.02	312.75
60	0	0	0	0	1000.04	590.43		16.9650	2014.79	282.82

Table 3 reports the raw readings values from the tests performed: V-funnel teste (t-funnel), flow test (D-flow1, D-flow2), compressive strength test at the age of 24 h (f_c_^24h^1.1, f_c_^24h^1.2, f_c_^24h^2.1, f_c_^24h^2.2, f_c_^24h^3.1, f_c_^24h^3.2) and the compressive strength test at the age of 28 d (f_c_^28d^4.1, f_c_^28d^4.2, f_c_^28d^5.1, f_c_^28d^5.2).

**Table 3:** Readings from tests.

Run Order	t-funnel [s]	D-flow1 [mm]	D-flow2 [mm]	f_c_^24h^1.1 [MPa]	f_c_^24h^1.2 [MPa]	f_c_^24h^2.1 [MPa]	f_c_^24h^2.2 [MPa]	f_c_^24h^3.1 [MPa]	f_c_^24h^3.2 [MPa]	f_c_^28d^4.1 [MPa]	f_c_^28d^4.2 [MPa]	f_c_^28d^5.1 [MPa]	f_c_^28d^5.2 [MPa]
1	18.44	34.2	34.2	60.87	56.94	60.28	61.84	60.33	61.05	106.06	109.04	109.07	108.45
2	29.31	31.2	30.3	53.68	54.22	52.84	53.07	55.88	53.90	105.26	104.66	100.80	96.38
3	11.78	37.4	38.0	53.05	53.40	53.10	54.55	54.07	52.04	108.67	109.04	111.70	106.69
4	30.84	31.6	31.9	54.97	54.39	55.60	55.44	56.45	56.41	108.84	104.38	104.10	108.11
5	108.22	23.0	22.8	62.06	61.12	61.45	61.22	63.52	59.15	104.39	105.58	104.06	102.40
6	18.00	33.8	34.3	54.95	56.37	56.58	55.84	54.13	56.33	106.73	107.74	108.01	109.36
7	15.46	35.3	36.8	57.84	59.90	61.95	59.97	63.13	60.72	115.36	115.17	112.36	115.17
8	12.78	37.3	36.6	54.18	56.78	56.57	54.22	54.91	56.75	106.10	106.70	102.70	105.07
9	162.22	31.6	30.2	62.07	63.08	62.16	62.44	62.59	64.86	113.19	105.17	111.71	114.83
10	20.47	33.4	34.4	56.88	58.68	57.95	58.42	59.65	60.63	105.92	110.92	107.38	104.70
11	18.91	34.4	34.3	53.50	54.07	56.50	56.65	53.03	53.55	104.20	104.74	107.88	110.57
12	14.75	35.9	35.9	57.78	57.73	57.23	57.77	58.70	57.00	108.66	118.61	114.67	116.54
13	23.56	32.6	33.0	59.18	60.83	61.62	60.67	63.10	64.58	106.60	114.39	113.52	111.18
14	21.83	32.7	32.3	59.29	65.52	63.42	61.58	63.28	62.97	116.97	113.68	113.25	119.26
15	18.25	33.4	32.9	57.91	58.90	58.18	57.87	59.30	58.98	113.20	111.53	110.04	111.83
16	20.91	32.3	32.7	62.97	63.26	61.66	60.82	63.18	64.49	115.03	105.60	117.78	110.05
17	29.28	30.5	30.3	61.22	62.05	63.72	60.67	60.41	63.71	114.37	112.82	114.03	108.79
18	17.59	34.1	34.3	56.21	57.13	57.08	54.15	57.28	59.54	114.37	109.83	109.32	115.18
19	12.51	36.6	37.0	52.57	60.10	59.16	59.77	59.75	57.80	118.77	116.15	116.32	111.80
20	16.66	34.5	35.0	54.56	56.87	57.00	55.43	55.82	57.09	114.31	116.66	109.92	114.22
21	16.16	34.4	34.6	51.07	51.90	53.20	50.97	49.91	50.78	111.85	107.24	100.36	104.52
22	45.44	27.0	26.9	64.40	61.33	65.47	61.40	63.33	61.37	101.49	104.86	103.41	105.86
23	17.12	34.6	35.2	55.04	56.71	57.66	56.75	56.60	57.70	109.92	113.20	119.93	118.64
24	21.38	32.9	32.9	56.68	56.48	59.87	58.57	58.70	60.04	117.30	115.18	123.36	114.91
25	18.82	33.4	34.1	58.86	60.10	61.17	59.75	56.64	58.57	126.29	121.43	111.29	111.59
26	13.03	35.3	36.2	56.13	55.70	56.73	59.12	58.37	58.05	113.77	112.58	116.55	113.04
27	39.62	30.6	30.6	61.84	58.37	60.74	60.95	58.52	61.76	113.43	110.78	113.79	116.04
28	52.16	28.2	28.2	57.71	58.93	59.29	60.22	58.11	58.52	103.36	107.22	113.08	116.51
29	12.38	37.0	37.0	58.72	59.05	57.26	57.09	56.61	56.36	110.68	114.47	116.27	117.05
30	23.47	33.8	33.8	61.08	62.72	61.75	61.23	61.90	61.66	n.av.	n.av.	n.av.	n.av.
31	18.28	35.1	35.3	55.85	55.98	56.75	54.26	55.57	56.05	106.27	107.04	111.46	112.10
32	34.03	31.6	32.0	53.41	50.62	52.59	51.26	52.90	53.41	104.61	102.29	102.18	100.71
33	11.45	36.7	36.7	50.64	51.02	49.36	49.14	52.73	52.37	106.14	101.94	114.89	104.14
34	35.50	31.2	31.7	50.90	51.96	50.49	49.20	48.54	48.89	95.59	100.99	96.59	98.41
35	60.25	30.1	31.0	53.49	56.47	55.38	53.21	53.40	55.84	103.02	97.49	104.74	99.79
36	17.69	34.0	34.2	51.21	49.87	51.28	52.86	53.42	51.17	105.78	106.81	107.84	104.15
37	18.34	33.6	34.4	54.62	54.88	55.35	53.92	55.35	53.04	105.40	106.65	103.56	110.42
38	16.13	36.4	36.4	58.69	59.89	59.84	61.46	58.82	58.77	116.53	110.91	107.91	111.26
39	12.07	37.6	37.6	52.91	52.32	51.66	53.31	51.06	52.57	110.13	107.75	109.62	102.00
40	43.09	31.2	32.0	59.02	58.74	59.58	59.32	58.87	59.34	104.22	103.29	101.46	103.54
41	19.71	33.3	33.6	53.87	53.53	53.23	52.70	52.85	56.41	101.71	100.83	102.51	105.11
42	16.25	34.8	34.8	51.11	53.17	50.40	52.86	52.43	53.01	106.15	103.06	105.60	104.67
43	26.37	32.7	33.5	54.92	58.51	56.08	57.85	55.89	56.14	107.33	97.55	107.86	102.38
44	30.50	33.1	33.1	56.21	58.45	59.41	62.70	60.24	59.93	105.75	110.20	110.46	107.56
45	35.44	32.8	32.5	59.16	56.30	62.86	59.48	60.43	61.73	109.91	115.86	111.56	102.81
46	27.29	33.3	33.2	59.11	60.94	58.63	62.41	61.10	63.01	113.66	107.28	112.67	108.88
47	32.23	31.7	31.8	55.60	55.38	55.28	54.70	55.29	56.31	101.36	106.22	105.68	103.58
48	22.41	33.1	33.2	52.66	50.29	53.71	52.26	53.02	51.26	108.18	106.79	106.21	104.50
49	27.50	33.3	33.6	51.51	52.94	52.99	52.66	54.50	52.66	108.30	105.97	106.25	109.50
50	19.59	35.2	35.6	55.32	54.49	56.80	51.65	54.86	54.93	101.38	108.30	107.56	109.33
51	19.82	34.3	34.4	48.06	48.82	46.12	49.50	49.91	47.34	98.57	98.96	97.13	99.93
52	42.10	31.1	31.4	64.05	62.79	61.66	65.50	63.64	66.15	106.89	108.83	110.32	104.87
53	22.85	33.2	33.3	54.98	56.91	53.97	55.94	54.70	55.56	105.25	103.85	102.60	107.11
54	26.47	32.7	32.7	58.70	55.03	54.88	52.92	57.55	58.35	106.72	106.87	107.06	104.96
55	23.00	33.0	33.3	58.32	56.05	57.60	56.16	55.90	n.av.	108.25	109.93	106.89	106.78
56	15.18	35.7	35.8	54.89	55.69	56.14	56.20	55.60	56.10	102.43	103.00	102.73	106.84
57	64.50	31.1	31.4	51.84	53.27	43.26	53.82	53.87	56.37	102.03	100.69	103.18	100.05
58	66.78	29.0	28.8	51.95	53.89	55.24	52.97	56.11	57.09	97.63	103.57	101.34	101.67
59	14.25	36.6	36.6	51.07	55.59	51.61	53.57	53.17	53.56	102.92	96.70	103.99	105.92
60	22.87	34.1	33.5	53.43	54.01	56.21	54.77	54.21	53.89	106.08	106.84	105.81	109.66

n.av.: The reading value is not available due to problems in the press equipment.

## 2 Experimental Design, Materials and Methods

In this experimental program two powders, two superplasticisers and one sand were used. The powders were the cement CEM I 42.5 R (EN 197-1 [1]) and the limestone filler, the specific gravity being 3.1 g/cm^3^ and 2.68 g/cm^3^, respectively. The sand was the normensand (EN 196-1 [2]) with specific gravity of 2.63 g/cm^3^ and water absorption of 0.30%. The superplasticizers were from third generation, polycarboxylate based, one (A) with ρ=1.08 g/cm^3^ and solid content of 40% and another (B) with ρ=1.07 g/cm^3^ and solid content of 30%. Distilled water was used.

The mix proportions were determined for a batch with a volume of 1.61 L. The design of experiments was defined for a 2^4^ factorial design consisting of 16 treatment combinations augmented by 8 axial runs plus 6 central runs, resulting in a central composite design with a total of 30 mortar trial mix composition. Additionally, due to the qualitative independent variable an extra central composite design was carried out. Mix proportions were calculated based on the four independent design variables: Water_v_/Cement_v_, Superplasticyzer_m_/Powder_v_, Water_v_/Powder_v_, Sand_v_/Mortar_v_. Each independent variable was evaluated at the levels -α, -1, 0, +1, +α, with α =2.0. The equivalence between the coded values and the real values are presented in the Table 1.

The following procedure was done to produce mixes: (i) the constituent materials were previously weighed and stored in plastic containers; (ii) in a standard mixer for mortars [2], 80% of the total water was joined to the powders and the sand (that moment was defined as being *t* = 0), and then the constituent materials were mixed at low speed for 120 s; (iii) stop mixing for 60 s to clean the paddle and (iv) add the superplasticiser and the remaining water in the last 10 s; (v) re-started mixing at low speed and mix for 120 s; (vi) stop mixing for 30 s to clean the paddle; (vii) re-started mixing at high speed and mix for 30 s.

Just after mixing the V-funnel test and the slump test were performed. From these tests, the reading of the flow time (t-funnel – see Ref. [3] for details) and the readings of the two orthogonal distances of the slump test (D-flow1, D-flow2 – see Ref. [3] for details) were taken. After that, five prismatic specimens 4 × 4 × 16 [cm] were moulded. These five specimens were stored in a climatic chamber at 20 °C.

All the specimens were unmoulded at the age 23.5 h. The compressive strength test was carried out in three specimens at the age of 24 h (with 15 min tolerance) and the others two specimens were stored in a climatic chamber at 20 °C and at least 98% of RH up to the age of 28 d. At the age of 28 d the last two specimens were tested to the compressive strength, too. Note that, previously to the compressive strength test, the tensile strength test [2] was done in the specimens to break the specimens in two halves (no values were recorded). Immediately after that, the halves were tested to the compressive strength [2] (f_c_^24h^1.1, f_c_^24h^1.2, f_c_^24h^2.1, f_c_^24h^2.2, f_c_^24h^3.1 and f_c_^24h^3.2) at the age of 24 h and (f_c_^28d^4.1, f_c_^28d^4.2, f_c_^28d^5.1 and f_c_^28d^5.2) at the age of 28 days.

## Ethics Statements

Nothing to declare.

## CRediT Author Statement

**Lino Maia:** I did all the research work. No other scientific contributions.

## Declaration of Competing Interest

The authors declare that they have no known competing financial interests or personal relationships that could have appeared to influence the work reported in this paper.
